# Supplementary material for: Effects and functional mechanisms of digital fitness platforms on online fitness payment behavior under the perspective of “She-economy”
Source: PLoS One. 2025 Mar 10;20(3):e0319246. doi: 10.1371/journal.pone.0319246 (PMC11892851; doi:10.1371/journal.pone.0319246)
Supplement: Attachment — Submitted filename: Response to Reviewers.doc [file pone.0319246.s002.doc]

**Journal Requirements:**

Q1: Please ensure that your manuscript meets PLOS ONE's style requirements, including those for file naming.

**Response:** Thank you for this comment, and we have rearranged our manuscript to meet PLOS ONE's style requirements, including those for file naming.

Q2: You indicated that ethical approval was not necessary for your study. We understand that the framework for ethical oversight requirements for studies of this type may differ depending on the setting and we would appreciate some further clarification regarding your research.

**Response:** Thank you very much for your advice. I have submitted a certificate from my institution certifying that this study does not require ethical approval.

Q3: In the online submission form, you indicated that "Data will be made available on request."All PLOS journals now require all data underlying the findings described in their manuscript to be freely available to other researchers. either 1. In a public repository, 2. Within the manuscript itself, or 3. Uploaded as supplementary information.
**Response:** Thank you very much for your comment. I have uploaded the data as supplementary information.

Q4: PLOS requires an ORCID iD for the corresponding author in Editorial Manager on papers submitted after December 6th, 2016. Please ensure that you have an ORCID iD and that it is validated in Editorial Manager.

**Response:** Many thanks for this review. The corresponding author is registered with ORCID iD and has been verified in Editorial Manager.

**Reviewer #1:**

Q1: The article explores the consumer market trends and behaviors in the context of women's fitness, emphasizing the significant role of women in driving sports consumption. It discusses the evolving perceptions of women's fitness, the influence of national policies, and the impact of digital fitness platforms on women's fitness behaviors.The development and design of digital fitness platforms require a combination of individual characteristics and product innovation to meet the needs of different users. Perceived ease of use is the most basic feature, making it easy for users to get started. Additionally, perceived enjoyment is crucial for engaging female users and providing a more enjoyable fitness experience. Developers should focus on consolidating the ease of use of basic functions and continuously expanding the product field through brand co-branding and supply chain integration. Enhancing user retention through personalized fitness plans and community-building activities is essential for the long-term success of digital fitness platforms.

**Response:** Thank you very much for your comment. Indeed, the paragraph is not precise enough. I have therefore made the following changes in line with your comments:

The development and design of digital fitness platforms require a combination of individual characteristics and product innovation to meet the needs of different users. Perceived ease of use is the most basic feature of the platform, and they make it easy for users to get started. Additionally, perceived enjoyment is crucial for engaging female users and providing a more enjoyable fitness experience. Therefore, digital fitness platforms need to consolidate the ease of use of their basic functions and provide users with an efficient and convenient experience in terms of UI interface, navigation structure, and accessibility. On the other hand, we also need to continuously expand the product field through brand co-branding and supply chain integration, continuously update the product content, and use the platform's social features to organize group activities online and offline to enhance its enjoyment. Enhancing user retention through personalized fitness plans and community-building activities is essential for the long-term success of digital fitness platforms.

**Reviewer #2:**

Q1: While it can be intuitive, it is not clear what is exactly meant by “She-economy”. A definition is missing.

**Response:** Thank you for your comments and suggestions. We were indeed missing a definition of “her economy”. Therefore, we have added the following to this study.

She-economy

In 1999, Kathy Matsui and Goldman Sachs proposed a theory related to women's economy, namely ‘women's economics’, which was first published in the British magazine The Economist and was mainly used to illustrate the economic benefits that women could obtain in the workplace [42]. After more than 20 years of development, the connotation and extension of the ‘she economy’ have changed dramatically. Shi Qingqi's idea has gained consensus among academics, who believe that because of women's esteem for consumption and the remarkable effect of driving the economy, the unique economic circle and economic phenomenon that has formed around women's financial management and consumption is called “She-economy” [43]. This study likewise draws on the above viewpoints for the definition of “She-economy”. Currently, more businesses and industries have begun to define the consumer population from a women’s perspective and develop products based on the needs of women. Most economic experts are of the view that women's economic self-reliance, surging consumer demand, and purchasing power are constituting a new point of economic growth.

Q2: What do TAM (line 67), SEM and fsQCA (line 84) stand for?

**Response:** Thank you for your question. Indeed, the use of TAM (line 67) is not precise enough and SEM and fsQCA are not adequately described, which may lead to confusion. Therefore, we modified the description of TAM, SEM and fsQCA as follows:

Most of the studies on digital fitness platforms have used the determinants of TAM as the independent variable...

**Structural equation modeling**

Structural Equation Modelling (SEM) is an important statistical method for quantitative research in contemporary behavioural and social fields. It can identify, estimate and validate a variety of causal models, and has the advantage of completing the analysis of a complex model at one time, without having to split the model, and SEM has already become an obvious discipline in data analysis[54]. The common application for SEM is Amos.

In this study, SEM was applied to validate the hypothetical model diagram of the influential relationship between digital fitness platforms and online fitness payment behaviour.

**Fuzzy set qualitative comparative analysis**

Fuzzy set qualitative comparative analysis (fsQCA) is the incorporation of ideas from holism, group comparison theory, and set theory into the social science research context, examining the complex combinations of causes of social phenomena and the ways in which they influence them from a holistic and systemic perspective, aiming to uncover the combinations of antecedent conditions that lead to outcomes. In contrast to SEM, it distinguishes itself from both traditional qualitative research methods, which emphasise the interpretation, description, and prediction of individual cases or a small number of cases, and traditional quantitative research methods, which emphasise the statistical analysis of large samples of data, by exploring intermediate paths that circumvent the limitations of these two research methods [55]. There are three main types of qualitative comparative analysis methods, clear set qualitative comparative analysis (csQCA), multi-valued set qualitative comparative analysis (mvQCA), and fuzzy set qualitative comparative analysis (fsQCA). fsQCA avoids the limitations of csQCA and mvQCA, and does not need to take into account the degree of change of the research variables and the problem of partial affiliation. Therefore, fsQCA has received more attention in recent years in various research areas.

This study adopts the fsQCA method mainly for the following reasons: firstly, SEM-based empirical studies have verified the positive influence of digital fitness platforms on online fitness payment behaviour. However, this traditional quantitative research method is based on the assumption that the relationship between variables is linear, testing the net effect between variables, analysing the independent effects between various types of variables, and failing to analyse the combined effects between variables. The fsQCA method precisely studies the multiple causal relationships formed by different combinations of antecedent conditions from a group perspective, which provides a wider research paradigm on online fitness payment behaviour, and can, to a certain extent, complement SEM.

Q3: The citations in the text do not mention the year of publication. Sections are not numbered.

**Response:** Thank you for your suggestions regarding the citations in the article, but I apologise. Since PLOS uses the "Vancouver" style, which requires citing the reference number in square brackets, we did not add the year of publication in the in-text citations. Following your advice and the journal's requirements, we have listed the references at the end of the manuscript and numbered them in the order they appear in the text.

Q4: The SOR theory is not clearly explained, nor it is clear how it is used in the study.

**Response:** Thank you for your suggestions regarding the SOR theory. To make this study more rigorous and enhance the persuasiveness of the theoretical model, we have restructured the explanation of the SOR theory to make it clearer based on your suggestions and have included its application in the study.

Mehrabian, Russell first proposed the SOR theory, which consists of three dimensions, S (stimuli) - O (organism) - R (response), and the relationship between the three is that the stimulus affects the internal state of the individual, which in turn drives the individual response [44]. SOR theory has been widely used in the field of consumer behavior [45]. The model suggests that an individual's buying behaviour is induced by physiological and psychological factors within the consumer as well as stimuli from the external environment. Consumers are stimulated by various factors to produce psychological changes to generate purchase motives and ultimately implement purchase behaviour. In recent years, SOR theory has been gradually applied to explore the impact of digital platform usage on users' consumption intentions and behaviors. For example, Tian et al. explored the factors influencing users' continued purchasing intention for fashion products in social e-commerce and confirmed that perceived value has a significant positive effect on continued purchasing intention [46]. Li Xinying Constructs the Influence Mechanism of Short Video Platform Usage on Users' Purchase Intention Based on Information Quality Theory and SOR Theory [47].

In a similar vein, digital fitness platform use may also have some impact on online fitness payment behavior. This study adopts the SOR theory as a research framework, with the core concept that women's online fitness payment behaviour is triggered by a stimulus that stems from women's intuitive experience of using a digital fitness platform and the intrinsic physiological and psychological factors of change that occur under the impetus of this stimulus, which in turn leads to purchasing intentions and behaviours. Therefore, perceived ease of use, perceived usefulness, and perceived fun from the TAM model were used as the ‘S’ frame in this study, and the ‘O’ frame was introduced to indicate the psychological cognitive changes of women in terms of satisfaction and willingness to continue using. The ‘S’ and ‘O’ frames are used by women to generate avoidance or proximity behaviours toward online fitness, thus completing the ‘R’ response.

Q5: Not clear what is meant here: “women’s psychological cognitive changes to online fitness occur after they feel internal and external stimuli using digital fitness platforms, which in turn affects women’s attitudes, intentions, and behaviors in fitness consumption”.

**Response:** Thank you for your suggestions. We must apologize for the confused description in this section. Therefore, to be more concise and clear, we have rewritten this part according to your suggestions as follows：

Mehrabian, Russell first proposed the SOR theory, which consists of three dimensions, S (stimuli) - O (organism) - R (response), and the relationship between the three is that the stimulus affects the internal state of the individual, which in turn drives the individual response [44]. SOR theory has been widely used in the field of consumer behavior [45]. The model suggests that an individual's buying behaviour is induced by physiological and psychological factors within the consumer as well as stimuli from the external environment. Consumers are stimulated by various factors to produce psychological changes to generate purchase motives and ultimately implement purchase behaviour. In recent years, SOR theory has been gradually applied to explore the impact of digital platform usage on users' consumption intentions and behaviors. For example, Tian et al. explored the factors influencing users' continued purchasing intention for fashion products in social e-commerce and confirmed that perceived value has a significant positive effect on continued purchasing intention [46]. Li Xinying Constructs the Influence Mechanism of Short Video Platform Usage on Users' Purchase Intention Based on Information Quality Theory and SOR Theory [47].

Q6: This is not clear: TAM affects individuals’ satisfaction and willingness to continue using it. TAM is a model and should not affect anything; it might be used to explain some relationships.

**Response:** Thank you for your valuable suggestions and rigorous review. Indeed, the phrasing here was not accurate, so we have made the following revisions:

In the TAM, perceived ease of use positively affects perceived usefulness, and this relationship is stable [48,56]. These variables also affect individuals’ satisfaction [57] and continue using intention [58].

Q7: The 7 hypotheses feel redundant and repetitive. This should be simplified.

**Response:** Thank you for your suggestion. To explore the relationship and mechanisms between perception and behavior more thoroughly, we made comprehensive hypotheses based on relevant literature. However, some of the hypotheses were proved to be redundant and repetitive, and the description also needed to be clearer. Therefore, we have made the following revisions:

H1a: Perceived ease of use positively and significantly affects satisfaction.

H1b: Perceived ease of use positively and significantly influences continue using intention.

H1c: Perceived ease of use positively and significantly affects perceived usefulness.

H2a: Perceived usefulness positively and significantly affects satisfaction.

H2b: Perceived usefulness positively and significantly influences continue using intention.

H3a: Perceived enjoyment positively and significantly affects satisfaction.

H3b: Perceived enjoyment positively and significantly influences continue using intention.

Q8: The definition of online payment behavior (line 224) is not clear. The literature that follows does not clarify it. Is payment behavior just the willingness to pay for a product/service?

**Response:** Thank you for your advice. Indeed, we also consider that the definition of online fitness payment behavior in this study may be confused, and it should not merely represent payment intention. Therefore, based on your advice, we further clarified the concept of online fitness payment behavior as follows:

Fitness behaviour is people's purposeful and conscious use of free time under the interaction of intrinsic factors and the external environment, to take the form and means of physical exercise, with the aim of physical and mental health or to achieve physical activities that are beneficial to people's health [64]. Online fitness payment behaviour is the subjective activity of individuals who use the Internet to purchase fitness products and services in order to achieve fitness behaviour [65]. Consistency of purchase and recommendations to others are important indicators of consumer behaviour [66].

Q9: The hypotheses from 10 to 18 are redundant, unclear and it is not discussed where they stem from.

**Response:** Thank you very much for your valuable suggestion. Through detailed reading and discussion, we also consider that hypotheses 10-18 are indeed redundant and make the article less clear and concise. Therefore, based on your suggestion, we believe it is will be appropriate to remove them.

Q10: Fig 1: everything seems to affect directly and indirectly anything else…

**Response:** Thank you for your valuable recommendation. We also believe that Fig1may mislead the readers’ understanding of the article. This study aims to deeply explore the relationship between digital fitness platforms and online fitness payment behavior under the perspective of the “She Economy.” Based on previous research, we established a relatively complex model and proposed corresponding hypotheses. However, there were indeed too many hypotheses. Therefore, based on your suggestion, we have removed some of the hypotheses. Additionally, to make the chart simpler and more intuitive, we have simplified and modified the chart.

Q11: The data analysis should be better described and the results better explained and discussed. It is unclear whether the research questions presented in the introduction have been answered.

**Response:** Thank you very much for your suggestion. Indeed, the explanation of conclusion and discussion was not well described and explained. Therefore, based on your suggestion, to enhance the depth and practical significance of this study, we have restructured the research conclusions according to the questions raised in the introduction.

Based on the SOR theory, the study explored the mechanisms and pathways influencing women's online fitness payment behaviour through SEM and fsQCA, and drew the following key conclusions to answer the three questions posed in the introduction.

（1）The use of digital fitness platforms in the context of the ‘she-economy’ affects individual online fitness payment behaviour. First, from the ‘S’ dimension, women's perceived use of digital fitness platforms is positively related to satisfaction、 continue using intention, except for the relationship between perceived usefulness and satisfaction, which is not significant. This suggests that the perceived ease of use of digital fitness platforms in the perspective of ‘She-economy’ positively affects the perceived usefulness, individual satisfaction, and continue using intention; perceived usefulness positively affects individual continue using intention, but does not affect individual satisfaction. Perceived enjoyment positively affects individuals' satisfaction and continue using intention. Second, from the ‘O’ level, satisfaction, continue using intention and online fitness payment behaviour are positively correlated, indicating that individuals' satisfaction or continue using intention positively affects their online fitness payment behaviour. It can also be found that the mean value of perceived enjoyment is the smallest, but the influence path coefficient is higher, indicating that perceived enjoyment is a perceived experience that women are more concerned about, but the use of digital fitness platforms does not meet the expectations of all women, which requires developers to consider how to improve the enjoyment of digital fitness platforms when designing the platform.

The relationship between perceived usefulness and satisfaction was not significant. The author believes that there are the following reasons: firstly, from a physiological point of view, girls have lower extrinsic explosiveness as well as lower intrinsic testosterone hormones than boys, resulting in less efficient fitness and muscle building than boys, and thus less positive incentives and a lag in perceived usefulness of the platform. Secondly, the explanation based on compensation theory is that when women do not feel that digital fitness platforms can improve their quality of life, they become frustrated and look for new ways to make up for the loss, and enjoy feelings appear to make up for the lack of usefulness, thus creating compensation. The author’s pre-survey period found that more than 80 percent of women are very interested in Keep software medals, ‘can not be thin is not important, but the co-branded Cherry Pills medals I am bound to get, and I hope that in the future I can come out of the co-branded models of my idol, Wang X Kai,’ said the above interview from a master’s degree student in the direction of the sports industry, B. It can be seen that there is a compensatory mechanism between the enjoyment and usefulness of the platform.

（2）The mechanisms by which digital fitness platforms influence online fitness payment behaviours in the context of the ‘she-economy’ are as follows: first, perceived ease of use, perceived usefulness, and perceived enjoyment indirectly influence an individual's online fitness payment behavior through the mediating role of continue using intention. This suggests that useful, interesting, and easy-to-use digital fitness platforms can indirectly increase women's consumption on the platforms by extending their stay on the platforms and providing more traffic for the platforms to push their content, which is consistent with the findings of Song H G et al. [80] and Hong X et al. [81] Perceived ease of use indirectly influences individuals' online fitness payment behavior through the mediating role of satisfaction. This suggests that easy-to-use platforms can increase women's excitement and interest and generate satisfaction ratings, which in turn generates more purchasing behaviors. Perceived ease of use indirectly influences an individual's online fitness payment behavior through the mediating role of satisfaction and continue using intention. This is consistent with the explanation above that women develop positive attitudes towards them, which in turn increases the amount of time and money spent on them. Similar results have been found in previous studies, where the more positive consumers' attitudes were, the more time and money they spent [82].

Second, perceived enjoyment can positively influence individual satisfaction or continue using intention, promoting individual online fitness payment behavior, but the mediating effect is not significant. The author argues that in the use of digital fitness platforms, women may have impulse spending due to a strong interest in the platform, without overly focusing on satisfaction and continue using intention.

（3）There are three types of conditional variable groups that can explain online fitness payment behaviour. First, the ‘hedonic’ path suggests that women are more concerned with the ability of digital fitness platforms to deliver a pleasurable and enjoy experience and focus on their own attitudes towards digital fitness platforms. The ‘integrated’ path indicates that women focus on a diversified and all-rounded experience, and they hope that digital fitness platforms can provide multi-element and multi-component courses and activities to meet different needs and interests. The ‘pragmatic’ path has the lowest coverage, suggesting that a small number of women in the ‘she-economy’ are only interested in the usefulness of digital fitness platforms and not in the other technologies behind them. Research in social psychology says that women’s physical attractiveness plays an important role in social behavior [83], and this pragmatic attitude may stem from women’s immediate need for physical attractiveness, which is more focused on practical results and utility.

Second， ‘Integrated’ digital fitness platforms are the most popular and favourite among women, followed by ‘hedonic’ and finally ‘pragmatic’. The study concludes that women’s current perceived ease of use of digital fitness platforms largely meets their usage needs, but perceived enjoyment and perceived usefulness do not. This requires that developers consider, in future designs, how to enhance the digital fitness platform to help women’s lives, increase their interest in using it, and fulfill their online fitness needs.

Q12: Overall, and looking at the title, it is not easy to understand what the contribution and the objectives of the paper are.

**Response:** Thank you very much for your comments. Indeed, the title of this study needs to correspond more clearly to the content of the study. Therefore, this study has made substantial changes to the content of the study, so please take the trouble to review my paper again. It is because of your valuable comments that this study has been improved, so thank you again for your hard work reviewing it. At the same time, the contributions and objectives of this study were not expressed clearly enough in the text, so the following revisions have been made anew:

The purpose of this study is to (1) investigate whether the use of a digital fitness platform influences their online fitness payment behavior; (2) if so, what are the specific functional mechanisms of the effect; (3) What combinations of conditional variables explain online fitness payment behavior. The novelty of this study mainly includes the following three aspects: First, there are few studies on women's sports consumption. This study can explore the mechanism of digital fitness platform's influence on online fitness payment behaviour from the perspective of ‘She-economy’, thus enriching related research. Second, most of the previous studies explored the influencing relationship between variables in a quantitative way and proposed paths in a qualitative way, while this study used two quantitative analysis methods, SEM and fsQCA, to analyze the influencing mechanisms and paths of digital fitness platforms and online fitness payment behaviours, which increased the scientific validity of the study. Third, the introduction of perceived enjoyment in this study further enriches the TAM and extends the application of SOR theory to women’s consumption.

**Reviewer #3:**

Q1: A literature search provides not only an opportunity to learn more about a given topic but provides insight on how the topic was studied by previous analysts. It helps to interpret ideas, detect shortcomings and recognize opportunities. In short, systematic and well-organized research may help in designing a novel research.

**Response:** Thank you for your comments. We all truly appreciate your encouragement. Thank you again for your support!
