# Supplementary material for: Effects and functional mechanisms of digital fitness platforms on online fitness payment behavior under the perspective of “She-economy”
Source: PLoS One. 2025 Mar 10;20(3):e0319246. doi: 10.1371/journal.pone.0319246 (PMC11892851; doi:10.1371/journal.pone.0319246)
Supplement: Attachment — Submitted filename: Response_to_Reviewers_auresp_2.doc [file pone.0319246.s003.doc]

**Reviewer #2:**

**Response:** Thank you for your support!

**Reviewer #4:**

**Q1:** While most of the previous reviewers' comments have been addressed, one recommendation—to number the sections—remains unimplemented.

**Response:** Thank you very much for your valuable comments. Indeed, we have carefully revised and improved the comments of the previous reviewers. However, regarding the suggestion of numbering the chapters, it is true that we have not yet implemented it, for which we deeply apologise. We have now numbered the sections to enhance the clarity and conciseness of the article.

**Q2:** Abbreviations:

The manuscript uses abbreviations without first providing their full forms. For instance, the first usage appears in the Abstract. The previous reviewer likely meant that you should introduce the full term before using the abbreviation, rather than including extensive explanations of the concepts.

**Response:** Thank you for your comments. I strongly agree with your comments, so I have rechecked this study to align the abbreviations in the manuscript into line with the requirements for their use and to remove unnecessary explanations.

**Q3:** Literature Review:
Lines 84-85: The statement "there are few studies on women’s sports consumption" is debatable, as I have come across a number of studies in this area. It would be beneficial to clarify why the existing studies are deemed insufficient.
Lines 87-89: Consider providing specific examples of studies to support your statements.
Lines 125, 190: It would strengthen your argument to cite relevant studies here as well.

**Response:** Thank you very much for your advice, and I fully agree with your comments.. Indeed, my presentation was not precise enough. Therefore, I have reorganised this section in lines 84-85. In lines 87-89, I have added specific research examples to support my statement. Additionally, relevant studies have been cited in lines 125 and 190 to further strengthen the discussion. The following changes have been made:

Lines 84-85: First, there is a lot of existing research on women's sports consumption, but only a small number of studies have focused on women's sports consumption under the influence of digital technology [28], and even fewer have studied the impact of women's online sports consumption behaviour.

Lines 87-89: For example, Chung K et al. [29] explored how the sensory experience of VR viewers affects their intention to consume VR products and services using only confirmatory factor analysis, and based on the above results, a path discussion was conducted.

Lines 125: Although other countries have earlier conducted research on women's fitness based on consumer perspectives [40].

Lines 190: TAM has yielded few results in the field of women's fitness [52].

**Q4:** Writing Style:

Lines 167-168: Ensure that only the appropriate words are capitalized. Currently, it seems that words are unnecessarily capitalized, which might be incorrect.

**Response:** Thank you for your comment and I apologise for the oversight. I have now fixed the case of this part of the word.

**Q5:** Methods:

It is not clear how many questions were included in the questionnaire. This should be specified.

Lines 318-324: The source of the questionnaire sets should be clearly cited in square brackets, not just by mentioning the author. For instance, the citation "from Jarvenpaa and Others" is unclear—if "Others" is not a name, it should be lowercase, and typically, multiple authors are referred to as "Jarvenpaa et al." The source should also be included in the references list.

**Response:** Thank you very much for your suggestion and I strongly agree with you that it is necessary to clarify the number of questions in the questionnaire and provide the source. Therefore, I have added the number of questions and cited relevant references to clarify the source of the questionnaire, as modified below:

The first part is the respondent's basic personal information, which consists of four questions that include demographic characteristics such as age and education. The second part was the measurement of the variables of interest, which consisted of 18 questions. The research team followed the principles mentioned in the study of Eisinga R et al. [78]. Poor quality items had to be removed from the limited pool of items, and the number of items in the scale could be two, as long as it was ensured that we were assuming that the available data were reasonable. The six items measuring perceived ease of use and perceived usefulness were taken from the Davis F D et al. [51] TAM scale. The four items for perceived enjoyment were taken from Sun Q et al. [54] Scale. The two items for satisfaction were borrowed from Wen X et al.[68] scale. The three items for continuing using intention were borrowed from Lin J S C et al. [79] scale, and the three questions on online fitness payment behaviour are from the Jarvenpaa et al. [80] and Liu L et al. [81] scale.

**Q6:** Results and Discussion:

The results appear somewhat intuitive. It would be useful to elaborate on the practical and theoretical implications of your findings. This will help readers understand the significance of your research.

The number of valid questionnaires analyzed is 259, not 323. I think this number should be mentioned in the Abstract.

The manuscript is somewhat wordy in places. Consider streamlining the text for clarity and conciseness.

The discussion section is underdeveloped, with results being compared to only three previous studies. A more thorough comparison with existing research is needed.

**Response:** Thank you very much for your comment. I couldn't agree with you more.

(1) The results are indeed somewhat intuitive and it does help to elaborate on the practical and theoretical implications of the findings. Therefore, I first provided a detailed explanation of the research findings, and then added a revelation section at the end of the manuscript, where the theoretical and practical implications of the study were elaborated. The revisions are as follows:

## 7.1 Implications

In terms of theoretical implications. Firstly, this study illustrates how women's use of digital fitness platforms influences their online fitness payment behaviour, which will help us to understand women's online consumption behaviour more comprehensively. Second, this is a new application of SOR theory and an extension of current research on TAM. Women will feel good about a digital fitness platform that is enjoyment, useful, and simple to operate, and will want to continue to use it, thus spending money on that platform [101]. That is, women's perceptions of what digital fitness platforms will do affect their satisfaction and continue using intention them, which in turn affects their online fitness payment behaviour. However, it may be due to reasons such as women's presentation of fitness results being poorer than men's [102], thus producing a non-significant result between usefulness and satisfaction. Meanwhile, women are prone to paying for their interests and hobbies [103] and may spend impulsively to catch up with trends, leading to a less significant mediating role of satisfaction, and continue using intention between perceived enjoyment and online fitness payment behaviour. Third, this study used fsQCA, a quantitative analysis method, to explore the pathways. The results revealed three configurations of group pathways. “Integrated” is the dominant pursuit of women. In other words, women seek platforms that provide “fitness benefits + emotional value”. This was followed by “hedonic” and “pragmatic”.

In a practical sense, the development of platforms needs to enhance their attributes. First of all, perceived ease of use is the most basic function of the platform, and they can make it easy for users to get started. Therefore, platform developers and administrators need to consolidate the ease of use of their own basic functions and provide users with efficient and convenient experiences in terms of UI interface, navigation structure and accessibility. Secondly, perceived usefulness is the embodiment of the core functions of the platform, which can satisfy the initial intention of users to use it. On the one hand, artificial intelligence technologies such as big language models are actively applied. In the initial stage, the mining of user data is strengthened to accurately locate user needs and provide users with more personalized services; in the middle stage, the user data is trained on its own large model to provide users with more comprehensive body diagnosis and sports training planning; in the later stage, through the detailed analysis of the user's fitness characteristics and fitness habits, it is recommended for the user to recommend a more scientific, reasonable and diversified fitness In the later stage, through the detailed analysis of the user's fitness characteristics and fitness habits, we recommend more scientific, reasonable and diversified fitness programmes and suggestions for users. On the other hand, user retention should be fully considered to improve the user stickiness of the product. In the initial stage, the initial accumulation of customer base can be achieved through simplifying the registration process, guided experience and free trial period of courses, equipment coupons, and incentives for reaching the target; in the middle stage, the customer base can be cultivated through personalized fitness plans, customized training courses, comment and praise interaction, and regular discounts; in the late stage, the long-term customer base can be enhanced through continuous improvement, optimization of functionality, return to rewards, community building, customer service, and other methods. Building, and customer service to enhance long-term retention.

The development of the platform also needs to emphasise exchanges and cooperation with other products, services, or resources. First, talent is the first resource for development. Therefore, cultivating talents who understand digital and sports is the first major event. Digital fitness platforms can realise the effective cycle of the three penetrating elements of education, talent and technology through policy support, school-enterprise cooperation and other means, jointly cultivate talents that meet their own needs and provide guarantees for the continuous growth of the technical team and the platform itself through working practices such as platform design and operation. Secondly, perceived enjoyment is an important element that women are concerned about, which can attract female users and provide a more enjoyable fitness experience. On the one hand, through brand co-branding, supply chain integration and other ways to expand the product field, continue to update the product content, and make use of the platform's social features to organise online and offline group activities to enhance its enjoyment. On the other hand, by keeping up with the trend, popular hotspots, and so on, we continue to provide product stories, provide users with more connotative products, and promote marketing. Third, cross-border circle-breaking cooperation should be deepened to improve the efficiency of platform usage. On the one hand, the platform can cooperate with professional fitness coaches, nutritionists, and rehabilitators to increase the usage rate of the platform, improve the exposure rate of knowledge providers, and innovate the platform's business model; on the other hand, the platform can also cooperate with sports apparel sales, cultural and creative product sales, and wearable social bloggers, to enrich the content of the platform, and to achieve the simultaneous development of such products through sports themes such as camping, marathon, and cycling.

(2) As you suggested, the reference to the number of analysed valid questionnaires as 259 in the abstract is necessary. Therefore, I have revised the number of analysed valid questionnaires to 259 in the abstract.
(3) The manuscript section does seem to be a bit lengthy. Therefore, I have reorganised this section by removing and revising the lengthy areas to make it clear and concise.
(4) The discussion section did seem inadequate. Therefore I have rewritten this section to ensure a more comprehensive comparison with existing research. The revisions are as follows:

## 6.1 Discussion

### 6.1.1 Influence relationships between variables

The core factors of TAM have a significant positive effect on the satisfaction, and continuing using intention of female users of digital fitness platforms, which in turn have a significant positive effect on online fitness payment behaviour. That is, the higher the attributes of the core factors of TAM, the stronger the satisfaction, and continuing using intention of the digital fitness platform for female users, which will further result in more consumption behaviours towards the digital fitness platform. This is consistent with Hasan’s [89] study coinciding with the fact that when women perceived online shopping to be less wise and less effective, women also had lower affective attitudes, including a preference for online shopping and feeling excited about it. From there, women also showed lower behavioural attitudes or behaviours. Therefore, as the digital economy and socio-cultural environment develops, the operability, usefulness, and enjoyment of digital fitness platforms will gradually increase and, women will increase their online fitness payment behaviour. This result also validates Richard et al. [90] findings on women's online consumer behaviour.

The perceived usefulness of only female users of digital fitness platforms does not have a significant impact on their satisfaction. The author believes that there are the following reasons: firstly, from the physiological point of view, girls’ external explosive power, as well as internal testosterone hormone, are lower than boys’, which leads to less efficient fitness and muscle building than boys', thus obtaining less positive incentives, and lagging in perceived usefulness for the software; secondly, based on the compensation theory for an explanation, when women do not feel that the digital fitness platform can improve the quality of life, they will have a sense of frustration, and look for new ways to make up for the loss, the enjoyment feeling appears just to make up for the lack of usefulness, thus forming compensation. During the author's pre-survey period, it was found that more than 70% of women were interested in the medals of Keep software, “It's not important whether I can lose weight or not, but I'm determined to get the co-branded medals of Cherry Mariko, and I hope that I can come out with the co-branded models of my idols in the future”, the above interview came from a master's degree student of the direction of the sports industry, B. It can be seen that there is a gap between the enjoyment and the usefulness of the platforms. There is a compensation mechanism between enjoyment and usefulness. Furthermore, women's consumption motives are easily influenced by factors such as fashion trends [91]. If they are involved in fitness to follow trends or keep up with their friends, the functionality and practicality of a digital fitness platform may not be their main concern, but more about whether the platform can provide a way to meet the current trends and thus satisfy their inner quest.

### 6.1.2 Mediating role of satisfaction, continue using intention

Continue using intention and satisfaction have mediating effects in the relationship between the core factors of TAM and online fitness payment behaviour, respectively. That is, useful, enjoyment, and easy-to-use digital fitness platforms can increase women's excitement, generate satisfaction ratings or prolong women's stay on the platform, provide more traffic to the platform's push content, and thus increase the amount of time and money women spend on the platform. This is consistent with the study by Sewak [92] and Gueguen et al. [93] that when female consumers receive vivid information and have pleasant experiences while using an app, they rate the app's communication higher or spend more time on the app, which can lead to increased consumption. This also follows the study by Le Mainardes, E.W. et al. [94] and Hong X et al. [95].

Perceived enjoyment can positively influence women satisfaction, which in turn promotes women online fitness payment behaviour, but the mediating effect is not significant. The author suggests that women may spend impulsively because of a strong excitement in the platform service, and are not overly concerned about satisfaction the digital fitness platform. This explains why some women impulsively spend money on signing up for contests or events on platforms just to win fun prizes.

### 6.1.3 The combined path of online fitness payment behaviour

The seven combinatorial variables of online fitness payment behaviour were classified into three types of constructs, namely “hedonic”, “integrative”, and “pragmatic”. The “hedonic” path suggests that women are more concerned with whether the services and products of digital fitness platforms can provide a pleasurable and enjoyment experience, as opposed to fitness outcomes. This further reflects the consumption characteristics of women [96], who tend to be easily attracted to services and products with novel and interesting designs when using digital fitness platforms. They may impulsively consume because of an interesting challenge activity, a personalised training plan or an attractive interface design on the platform. However, this kind of impulsive consumption may also bring some problems, such as they may buy services or products that are not suitable for them because of their momentary interest, leading to waste. Meanwhile, focusing too much on the hedonic experience may affect the achievement of the actual fitness effect to a certain extent. This is consistent with what Cheng Q [97] found. The “comprehensive” path indicates that women focus on diversified and all-round experience, and they hope that the digital fitness platform can provide multi-element and multi-element courses and activities to meet different needs and interests. Liu C et al. [98] believe that with the improvement of consumption ability and upgrading of consumption concepts, consumers are more inclined to buy high-quality, high-value and diversified goods. The “pragmatic” path has the lowest coverage rate, suggesting that a small number of women in the context of the “she-economy” are only concerned with the usefulness of digital fitness platforms but not the other technologies behind them. Social psychology research suggests that women's physical attractiveness plays an important role in social behaviour [99], and this pragmatic attitude may stem from women's direct need for physical attractiveness, with a greater focus on practicality and usefulness. Hargreaves J [100] argues that women work out to achieve the ideal body - slim, fit and youthful - and that the effort they put into achieving their goals.

**Q7:** Conclusion:

The conclusion section is too lengthy and should not include subtitles. Additionally, the practical and theoretical implications of the study are not clearly outlined. This is a critical element that needs to be addressed.

**Response:** Thank you for your suggestion regarding the conclusion section. We also believe that the conclusion section is too lengthy and should not include subtitles. At the same time, we consider that it is essential to clarify the practical implications and significance of the research. Therefore, I have firstly reorganised the conclusion section to ensure that the writing is up to scratch. Secondly, I added the revelation section to clearly outline the practical and theoretical implications of the study(this is elaborated in Q6 for specific modifications ). The revisions are as follows:

## 6.2 Conclusion

This study proposes a hypothetical model of digital fitness platforms on women's online fitness payment behaviours based on a literature review and using SOR theory. By analysing the survey data from 259 women, the following results were obtained: (1) Women's perceived ease of use of digital fitness platforms positively predicts perceived usefulness, satisfaction, and continue using intention, which in turn positively predicts online fitness payment behaviour. Satisfaction and continue using intention have a mediating role in this process, respectively. (2) Women's perceived usefulness of digital fitness platforms does not predict satisfaction, but positively predicts continue using intention, and thus positively predicts online fitness payment behaviour. continue using intention has a mediating role in this process. (3) Women's perceived enjoyment of digital fitness platforms can positively predict satisfaction and continue using intention, and thus positively predicts online fitness payment behaviour. but the mediating role of satisfaction and continue using intention is not significant. (4) There are 7 conditioned variable grouping paths that can explain online fitness payment behaviour, which can be classified into 3 types of “hedonic”, “integrative” and “pragmatic” according to the core and edge conditions.

**Reviewer #5:**

**Q1:** Comment 1 – The current title of the second section, "Research Basis", could be more informative. I suggest renaming this section to "Context and Theoretical Background." This revised title would better reflect the content, providing a clearer indication of what the reader can expect in this section.

**Response:** Thank you very much for your suggestion and I highly agree with your perspective. The revised title better reflects the content, so I have renamed this section ‘Context and Theoretical Background’.

**Q2:** Comment 2 – The subtitles "A Review of Research Related to Women’s Fitness" and "She-Economy" cover closely related themes and would benefit from being combined into a single subsection. I recommend a new title that highlights the integrated context of the research, such as "The Role of Women's Fitness and the She-Economy in Digital Consumer Behaviour." This would create a more cohesive narrative and emphasise the interconnectedness of these topics, allowing the reader to see the broader context of the study.

**Response:** Thank you very much for your comments and enlightened advice,. The use of a new title will synthetically highlight the context as well as emphasise the interconnections between these themes, which is necessary, so I have made the changes you suggested.

**Q3**: Comment 3 – I have observed that a previous reviewer requested clarification on the acronyms, "SEM" and "fsQCA", asking for their full forms. While this feedback highlights the need for clarity, it is not essential for the authors to provide detailed explanations of these methodologies within the second section of the manuscript. Instead, I recommend that the authors simply present the complete names of these methodological approaches the first time they are mentioned. For example, "SEM" as "Structural Equation Modelling" and "fsQCA" as "Fuzzy Set Qualitative Comparative Analysis."
These methodologies are well-established and widely recognised within the research community, and most readers familiar with the field will understand their significance without requiring extensive descriptions at this stage. Additionally, placing detailed methodological explanations in later sections where these methods are directly applied would enhance the manuscript’s structure and maintain the focus of the theoretical background. This approach will address the reviewer’s concern effectively while ensuring the manuscript remains clear and well-organised.

**Response:** Thank you for your suggestions, which provided me with a perfect way to make changes. Therefore, I first deleted this section and then added the application of these methods to the use section to enhance the structure of the manuscript and ensure that it is well organised.

**Q4:** Comment 4 – The second section of the manuscript provides a thorough review of the relevant literature, discussing key concepts such as women’s fitness, the She-Economy, and the associated consumer behaviours. While this review is informative, the section would greatly benefit from the inclusion of a conceptual framework that synthesises the reviewed literature and sets the stage for the subsequent hypotheses.
A conceptual framework is essential as it visually and theoretically links the key variables discussed in the literature review, providing a clear and structured foundation for the research. By introducing a conceptual framework after the literature review, the authors can more effectively illustrate the relationships between the different variables and concepts, such as how the She-Economy influences online fitness payment behaviour through variables like satisfaction, perceived ease of use, and perceived usefulness.
This framework should serve as a bridge between the literature review and the hypothesis formation, making the logical flow of the manuscript more evident to the reader. It would also enhance the overall coherence of the research by providing a clear, visual representation of the theoretical model that underpins the study.
I strongly recommend that the authors develop and include a conceptual framework at the end of the second section. This will not only strengthen the theoretical grounding of the research but also provide a clear roadmap for the hypotheses and the subsequent empirical analysis.

**Response:** Thank you very much for your insightful comment. Adding a conceptual framework indeed strengthens the visual and theoretical connection between the key variables discussed in the literature review, providing a clear and structured foundation for the research. Therefore, I have added a conceptual framework. The modifications are as follows:

## 2.4 The conceptual framework

In summary, the above provides an in-depth discussion of the role of women's fitness and the “She-economy” in digital consumer behaviour, as well as the theoretical basis of the “SOR theory” “TAM model”, the theoretical basis, the underlying context and the interrelationships between them. A well-constructed diagram (Fig 1) allows for a clearer and more organised presentation of these elements, enabling a more intuitive understanding of their connections and logical relationships, and providing a strong support for the following research to be carried out.

**Fig 1. The conceptual framework**

**Q5:** Comment 5 – The current section titled "Theoretical Assumptions" appears overly simplified, particularly in its approach to forming hypotheses. The section would benefit significantly from incorporating more deductive reasoning derived from the theoretical framework presented earlier in the manuscript. Currently, the hypotheses seem to be stated without sufficient grounding in the theoretical background, which weakens the logical flow and the connection between the literature review and the hypotheses.
To enhance this section, I recommend that the authors first revise the title to better reflect its content and purpose. A more appropriate title could be "Hypothesis Formation," which clearly indicates that this section is dedicated to the development of hypotheses based on the theoretical framework. This title change will help align the reader’s expectations and clarify the section’s role within the manuscript.
Moreover, in forming the hypotheses, it is crucial to draw more explicitly from the theoretical background. The authors should aim to demonstrate how each hypothesis logically follows from the theories and concepts discussed in earlier sections. For example, when introducing the hypotheses related to perceived ease of use, perceived usefulness, and perceived enjoyment, the authors should first discuss these concepts within the context of the Technology Acceptance Model (TAM) and related literature, thereby providing a stronger justification for each hypothesis. Similarly, when discussing satisfaction, continue using intention, and online fitness payment behaviour, the authors should reference relevant theories and prior studies to build a more robust foundation for the hypotheses.
By integrating these elements into the hypothesis formation process, the section will not only become more comprehensive but will also provide a clearer rationale for the proposed research model. This approach will significantly improve the overall coherence and scholarly rigor of the manuscript.

**Response:** Thank you very much for your suggestion. After thorough consideration, we also agree that this section could be more clear. Firstly, the previously presented conceptual framework lacked precision and was insufficient in supporting the hypotheses.. Therefore, according to your suggestion, we first formed a clear conceptual framework, and this is elaborated in Q4 for specific modifications. Secondly, the title has also been modified to better align with this framework, ensuring that it sets appropriate expectations for readers and clarifies the section’s role within the manuscript. Thirdly, I have referred to relevant theories and previous studies to reorganise the hypotheses so as to provide a more robust theoretical basis. The revisions are as follows:

# 3 Hypothesis Formation

## 3.1 TAM model and satisfaction, continue using intention

TAM is one of the most influential information systems acceptance models, which has been used to study the acceptance and behavioural patterns of different populations towards new technologies and products by analysing individuals' attitudes, perceived usefulness and perceived ease of use. A study by Li et al. [58] found significant gender differences in health information search between men and women. Then, there may be gender differences in the use of digital fitness platforms as a vehicle for health information search. Therefore, it is of great theoretical and practical significance to study the impact of digital fitness platforms on women's online fitness payment behaviours in the context of “She-economy”.

In the TAM model, perceived ease of use positively affects perceived usefulness and this relationship is stable [59]. The variables of TAM also affect individuals' satisfaction [60] and continue using intention [61]. Satisfaction is an expression of attitude that measures an individual's psychological response to a good, service, or experience, i.e., the difference between expectations and subjective feelings [62]. Continue using intention is a consumer's continued use of something rather than abandonment of its use. Using college students as research subjects, Zhan H [63] found that TAM affects male and female students' adoption of online teaching platforms to different degrees. Tan H et al. [64] further found significant differences between male and female students in the mechanism of satisfaction with online teaching and willingness to continue using it. Based on the TAM, Chen et al. [65] explored the behavioural characteristics and influencing factors of women's cross-border e-commerce consumption in China, and the study showed that the variables of the TAM had a significant impact on women's attitudes and willingness to cross-border e-commerce consumption. The TAM model of this study includes three determinants, perceived ease of use, perceived usefulness, and perceived enjoyment. Therefore, the following hypotheses are proposed:

## 3.2 Satisfaction, continue using intention and online fitness payment behavior

In the context of “She-economy”, the diversified development of Internet products and services has drawn attention to women's emotional experience on the Internet. Satisfaction is regarded as a very important emotional variable that can positively influence women intention and behaviour [66]. Fitness behaviour is women's purposeful and conscious use of free time under the interaction of intrinsic factors and the external environment, to take the form and means of physical exercise, with the aim of physical and mental health or to achieve physical activities that are beneficial to people's health [67]. Online fitness payment behaviour is the subjective activity of women who use the Internet to purchase fitness products and services in order to achieve fitness behaviour [68]. Consistency of purchase and recommendations to others are important indicators of consumer behaviour [69]. Low leisure time and low income are the two main reasons hindering women's fitness spending in China [70].

Women's satisfaction with a product or service is the main motivation for the continue using intention it [71], and an important factor influencing consumption behaviour [72]. In the original TAM model and SOR theory, attitude and continue using intention have an impact on actual behaviour. Lu Y et al. [73] prove the above point. Chen S et al. [74] explored the mechanism of single women's satisfaction on leisure tourism consumption behaviour based on SOR theory. Based on the TAM theory, Miao S [75] found that women's perceived usefulness and perceived ease of use of gaming platforms have a significant effect on consumption intention. Mailizar et al. [76] verified that college students' attitudes towards using e-platforms significantly affect their intention to use them. Reiter et al. [77] similarly prove the above point. Therefore, the following hypotheses are proposed:

**Q6:** Comment 6 – The discussion section of the manuscript, as it stands, is not sufficiently developed. It is important to emphasise that the discussion section plays a pivotal role in any research paper. This section is where the authors have the opportunity to showcase their critical thinking, derive solutions from their findings, and demonstrate a deeper understanding of the research problem. Currently, the discussion provided is largely descriptive and lacks the depth required to thoroughly engage with the implications of the findings.
To improve this section, the authors should focus on several key areas. First, the discussion should delve deeper into the broader implications of the findings. For instance, how do these results contribute to our understanding of women’s online fitness payment behaviour in the context of the She-Economy? What do these findings suggest for digital fitness platform developers, marketers, and policymakers? The discussion should not only reiterate the findings but also explore their significance in a broader context, comparing them with results from other studies and considering their practical applications.
Furthermore, the authors should engage more critically with the results, considering alternative explanations for the findings and discussing any limitations or unexpected outcomes. This critical analysis will demonstrate a more nuanced understanding of the research problem and provide a balanced view of the study’s contributions.
In summary, the discussion section should be expanded to include a more thorough analysis of the implications of the study, a comparison with other research, and a consideration of the broader impact of the findings. This will significantly enhance the manuscript’s overall quality and ensure that it provides valuable insights to the field.

**Response:** Thank you very much for your comments. Indeed, the discussion section of the manuscript was insufficient. Therefore, to enhance the quality of the manuscript, I have first reorganised the discussion section to highlight how the findings contribute to our understanding of women's online fitness payment behaviors within the context of the ‘she-economy’. Secondly, the findings are reiterated and their broader significance is explored by comparing them to previous research and considering alternative explanations for the results. Thirdly, in the Implications section, I elaborate on the theoretical and practical implications of the study to provide a clearer picture of the study's findings and elaborate on the implications of these findings for digital fitness platform developers, marketers and policymakers. The revisions are as follows:

## 6.1 Discussion

### 6.1.1 Influence relationships between variables

The core factors of TAM have a significant positive effect on the satisfaction, and continuing using intention of female users of digital fitness platforms, which in turn have a significant positive effect on online fitness payment behaviour. That is, the higher the attributes of the core factors of TAM, the stronger the satisfaction, and continuing using intention of the digital fitness platform for female users, which will further result in more consumption behaviours towards the digital fitness platform. This is consistent with Hasan’s [89] study coinciding with the fact that when women perceived online shopping to be less wise and less effective, women also had lower affective attitudes, including a preference for online shopping and feeling excited about it. From there, women also showed lower behavioural attitudes or behaviours. Therefore, as the digital economy and socio-cultural environment develops, the operability, usefulness, and enjoyment of digital fitness platforms will gradually increase and, women will increase their online fitness payment behaviour. This result also validates Richard et al. [90] findings on women's online consumer behaviour.

The perceived usefulness of only female users of digital fitness platforms does not have a significant impact on their satisfaction. The author believes that there are the following reasons: firstly, from the physiological point of view, girls’ external explosive power, as well as internal testosterone hormone, are lower than boys’, which leads to less efficient fitness and muscle building than boys', thus obtaining less positive incentives, and lagging in perceived usefulness for the software; secondly, based on the compensation theory for an explanation, when women do not feel that the digital fitness platform can improve the quality of life, they will have a sense of frustration, and look for new ways to make up for the loss, the enjoyment feeling appears just to make up for the lack of usefulness, thus forming compensation. During the author's pre-survey period, it was found that more than 70% of women were interested in the medals of Keep software, “It's not important whether I can lose weight or not, but I'm determined to get the co-branded medals of Cherry Mariko, and I hope that I can come out with the co-branded models of my idols in the future”, the above interview came from a master's degree student of the direction of the sports industry, B. It can be seen that there is a gap between the enjoyment and the usefulness of the platforms. There is a compensation mechanism between enjoyment and usefulness. Furthermore, women's consumption motives are easily influenced by factors such as fashion trends [91]. If they are involved in fitness to follow trends or keep up with their friends, the functionality and practicality of a digital fitness platform may not be their main concern, but more about whether the platform can provide a way to meet the current trends and thus satisfy their inner quest.

### 6.1.2 Mediating role of satisfaction, continue using intention

Continue using intention and satisfaction have mediating effects in the relationship between the core factors of TAM and online fitness payment behaviour, respectively. That is, useful, enjoyment, and easy-to-use digital fitness platforms can increase women's excitement, generate satisfaction ratings or prolong women's stay on the platform, provide more traffic to the platform's push content, and thus increase the amount of time and money women spend on the platform. This is consistent with the study by Sewak [92] and Gueguen et al. [93] that when female consumers receive vivid information and have pleasant experiences while using an app, they rate the app's communication higher or spend more time on the app, which can lead to increased consumption. This also follows the study by Le Mainardes, E.W. et al. [94] and Hong X et al. [95].

Perceived enjoyment can positively influence women satisfaction, which in turn promotes women online fitness payment behaviour, but the mediating effect is not significant. The author suggests that women may spend impulsively because of a strong excitement in the platform service, and are not overly concerned about satisfaction the digital fitness platform. This explains why some women impulsively spend money on signing up for contests or events on platforms just to win fun prizes.

### 6.1.3 The combined path of online fitness payment behaviour

The seven combinatorial variables of online fitness payment behaviour were classified into three types of constructs, namely “hedonic”, “integrative”, and “pragmatic”. The “hedonic” path suggests that women are more concerned with whether the services and products of digital fitness platforms can provide a pleasurable and enjoyment experience, as opposed to fitness outcomes. This further reflects the consumption characteristics of women [96], who tend to be easily attracted to services and products with novel and interesting designs when using digital fitness platforms. They may impulsively consume because of an interesting challenge activity, a personalised training plan or an attractive interface design on the platform. However, this kind of impulsive consumption may also bring some problems, such as they may buy services or products that are not suitable for them because of their momentary interest, leading to waste. Meanwhile, focusing too much on the hedonic experience may affect the achievement of the actual fitness effect to a certain extent. This is consistent with what Cheng Q [97] found. The “comprehensive” path indicates that women focus on diversified and all-round experience, and they hope that the digital fitness platform can provide multi-element and multi-element courses and activities to meet different needs and interests. Liu C et al. [98] believe that with the improvement of consumption ability and upgrading of consumption concepts, consumers are more inclined to buy high-quality, high-value and diversified goods. The “pragmatic” path has the lowest coverage rate, suggesting that a small number of women in the context of the “she-economy” are only concerned with the usefulness of digital fitness platforms but not the other technologies behind them. Social psychology research suggests that women's physical attractiveness plays an important role in social behaviour [99], and this pragmatic attitude may stem from women's direct need for physical attractiveness, with a greater focus on practicality and usefulness. Hargreaves J [100] argues that women work out to achieve the ideal body - slim, fit and youthful - and that the effort they put into achieving their goals.

## 7.1 Implications

In terms of theoretical implications. Firstly, this study illustrates how women's use of digital fitness platforms influences their online fitness payment behaviour, which will help us to understand women's online consumption behaviour more comprehensively. Second, this is a new application of SOR theory and an extension of current research on TAM. Women will feel good about a digital fitness platform that is enjoyment, useful, and simple to operate, and will want to continue to use it, thus spending money on that platform [101]. That is, women's perceptions of what digital fitness platforms will do affect their satisfaction and continue using intention them, which in turn affects their online fitness payment behaviour. However, it may be due to reasons such as women's presentation of fitness results being poorer than men's [102], thus producing a non-significant result between usefulness and satisfaction. Meanwhile, women are prone to paying for their interests and hobbies [103] and may spend impulsively to catch up with trends, leading to a less significant mediating role of satisfaction, and continue using intention between perceived enjoyment and online fitness payment behaviour. Third, this study used fsQCA, a quantitative analysis method, to explore the pathways. The results revealed three configurations of group pathways. “Integrated” is the dominant pursuit of women. In other words, women seek platforms that provide “fitness benefits + emotional value”. This was followed by “hedonic” and “pragmatic”.

In a practical sense, the development of platforms needs to enhance their attributes. First of all, perceived ease of use is the most basic function of the platform, and they can make it easy for users to get started. Therefore, platform developers and administrators need to consolidate the ease of use of their own basic functions and provide users with efficient and convenient experiences in terms of UI interface, navigation structure and accessibility. Secondly, perceived usefulness is the embodiment of the core functions of the platform, which can satisfy the initial intention of users to use it. On the one hand, artificial intelligence technologies such as big language models are actively applied. In the initial stage, the mining of user data is strengthened to accurately locate user needs and provide users with more personalized services; in the middle stage, the user data is trained on its own large model to provide users with more comprehensive body diagnosis and sports training planning; in the later stage, through the detailed analysis of the user's fitness characteristics and fitness habits, it is recommended for the user to recommend a more scientific, reasonable and diversified fitness In the later stage, through the detailed analysis of the user's fitness characteristics and fitness habits, we recommend more scientific, reasonable and diversified fitness programmes and suggestions for users. On the other hand, user retention should be fully considered to improve the user stickiness of the product. In the initial stage, the initial accumulation of customer base can be achieved through simplifying the registration process, guided experience and free trial period of courses, equipment coupons, and incentives for reaching the target; in the middle stage, the customer base can be cultivated through personalized fitness plans, customized training courses, comment and praise interaction, and regular discounts; in the late stage, the long-term customer base can be enhanced through continuous improvement, optimization of functionality, return to rewards, community building, customer service, and other methods. Building, and customer service to enhance long-term retention.

The development of the platform also needs to emphasise exchanges and cooperation with other products, services, or resources. First, talent is the first resource for development. Therefore, cultivating talents who understand digital and sports is the first major event. Digital fitness platforms can realise the effective cycle of the three penetrating elements of education, talent and technology through policy support, school-enterprise cooperation and other means, jointly cultivate talents that meet their own needs and provide guarantees for the continuous growth of the technical team and the platform itself through working practices such as platform design and operation. Secondly, perceived enjoyment is an important element that women are concerned about, which can attract female users and provide a more enjoyable fitness experience. On the one hand, through brand co-branding, supply chain integration and other ways to expand the product field, continue to update the product content, and make use of the platform's social features to organise online and offline group activities to enhance its enjoyment. On the other hand, by keeping up with the trend, popular hotspots, and so on, we continue to provide product stories, provide users with more connotative products, and promote marketing. Third, cross-border circle-breaking cooperation should be deepened to improve the efficiency of platform usage. On the one hand, the platform can cooperate with professional fitness coaches, nutritionists, and rehabilitators to increase the usage rate of the platform, improve the exposure rate of knowledge providers, and innovate the platform's business model; on the other hand, the platform can also cooperate with sports apparel sales, cultural and creative product sales, and wearable social bloggers, to enrich the content of the platform, and to achieve the simultaneous development of such products through sports themes such as camping, marathon, and cycling.

**Q7:** Comment 7 – The conclusion section of the manuscript, as currently structured, does not adhere to the standard format typically expected in academic research papers. Several elements within this section would be more appropriately placed in the discussion or in a separate section dedicated to "Implications and Limitations."
Firstly, the content under "Focus on perceived enjoyment," "Enhancing user retention," and "Improving technology development" delves into detailed recommendations and implications that are more suited for the discussion section. The discussion is where these insights should be thoroughly explored, considering how the study’s findings contribute to existing knowledge and offering practical recommendations for industry professionals. By relocating this content to the discussion, the authors can provide a more comprehensive analysis of how these findings align with or diverge from other studies, thereby offering a deeper understanding of the research problem.

Secondly, the "Shortcoming" subsection, which addresses the limitations of the study and suggests areas for future research, should be separated into its own distinct section titled "Implications and Limitations." This section is crucial as it provides transparency about the study’s constraints and guides future research efforts. By clearly delineating these points, the manuscript will present a more organised and coherent structure, making it easier for readers to navigate the conclusions and implications of the study.
The conclusion itself should focus on summarising the key findings of the research, highlighting the main contributions to the field, and succinctly stating the broader implications without going into detailed recommendations or limitations, which should be reserved for the other sections mentioned above.
In summary, I recommend reorganising the current conclusion section by transferring the detailed implications to the discussion, creating a separate "Implications and Limitations" section, and refining the conclusion to be a concise summary of the study’s contributions.

**Response:** Thank you again for your comments. I agree that the conclusions section did not adhere to academic conventions, and the title 'Shortcomings' was used inappropriately. Additionally, the recommendations and implications needed to be positioned more appropriately. Therefore, firstly, according to your suggestion, I have re-discussed the empirical results and stated the conclusions of the study. Secondly, I replaced the title ‘Shortcomings’ with ‘Implications and Limitations’, making it easier for readers to navigate the conclusions and implications of the study. Thirdly, to align with the standard academic format, I have extracted and refined the section on implications and limitations from the ‘conclusion’ section in the new manuscript and included it as a separate section that details both the theoretical and practical implications of the study.

## 6.2 Conclusion

This study proposes a hypothetical model of digital fitness platforms on women's online fitness payment behaviours based on a literature review and using SOR theory. By analysing the survey data from 259 women, the following results were obtained: (1) Women's perceived ease of use of digital fitness platforms positively predicts perceived usefulness, satisfaction, and continue using intention, which in turn positively predicts online fitness payment behaviour. Satisfaction and continue using intention have a mediating role in this process, respectively. (2) Women's perceived usefulness of digital fitness platforms does not predict satisfaction, but positively predicts continue using intention, and thus positively predicts online fitness payment behaviour. continue using intention has a mediating role in this process. (3) Women's perceived enjoyment of digital fitness platforms can positively predict satisfaction and continue using intention, and thus positively predicts online fitness payment behaviour. but the mediating role of satisfaction and continue using intention is not significant. (4) There are 7 conditioned variable grouping paths that can explain online fitness payment behaviour, which can be classified into 3 types of “hedonic”, “integrative” and “pragmatic” according to the core and edge conditions.

# 7 Implications and Limitations

## 7.1 Implications

In terms of theoretical implications. Firstly, this study illustrates how women's use of digital fitness platforms influences their online fitness payment behaviour, which will help us to understand women's online consumption behaviour more comprehensively. Second, this is a new application of SOR theory and an extension of current research on TAM. Women will feel good about a digital fitness platform that is enjoyment, useful, and simple to operate, and will want to continue to use it, thus spending money on that platform [101]. That is, women's perceptions of what digital fitness platforms will do affect their satisfaction and continue using intention them, which in turn affects their online fitness payment behaviour. However, it may be due to reasons such as women's presentation of fitness results being poorer than men's [102], thus producing a non-significant result between usefulness and satisfaction. Meanwhile, women are prone to paying for their interests and hobbies [103] and may spend impulsively to catch up with trends, leading to a less significant mediating role of satisfaction, and continue using intention between perceived enjoyment and online fitness payment behaviour. Third, this study used fsQCA, a quantitative analysis method, to explore the pathways. The results revealed three configurations of group pathways. “Integrated” is the dominant pursuit of women. In other words, women seek platforms that provide “fitness benefits + emotional value”. This was followed by “hedonic” and “pragmatic”.

In a practical sense, the development of platforms needs to enhance their attributes. First of all, perceived ease of use is the most basic function of the platform, and they can make it easy for users to get started. Therefore, platform developers and administrators need to consolidate the ease of use of their own basic functions and provide users with efficient and convenient experiences in terms of UI interface, navigation structure and accessibility. Secondly, perceived usefulness is the embodiment of the core functions of the platform, which can satisfy the initial intention of users to use it. On the one hand, artificial intelligence technologies such as big language models are actively applied. In the initial stage, the mining of user data is strengthened to accurately locate user needs and provide users with more personalized services; in the middle stage, the user data is trained on its own large model to provide users with more comprehensive body diagnosis and sports training planning; in the later stage, through the detailed analysis of the user's fitness characteristics and fitness habits, it is recommended for the user to recommend a more scientific, reasonable and diversified fitness In the later stage, through the detailed analysis of the user's fitness characteristics and fitness habits, we recommend more scientific, reasonable and diversified fitness programmes and suggestions for users. On the other hand, user retention should be fully considered to improve the user stickiness of the product. In the initial stage, the initial accumulation of customer base can be achieved through simplifying the registration process, guided experience and free trial period of courses, equipment coupons, and incentives for reaching the target; in the middle stage, the customer base can be cultivated through personalized fitness plans, customized training courses, comment and praise interaction, and regular discounts; in the late stage, the long-term customer base can be enhanced through continuous improvement, optimization of functionality, return to rewards, community building, customer service, and other methods. Building, and customer service to enhance long-term retention.

The development of the platform also needs to emphasise exchanges and cooperation with other products, services, or resources. First, talent is the first resource for development. Therefore, cultivating talents who understand digital and sports is the first major event. Digital fitness platforms can realise the effective cycle of the three penetrating elements of education, talent and technology through policy support, school-enterprise cooperation and other means, jointly cultivate talents that meet their own needs and provide guarantees for the continuous growth of the technical team and the platform itself through working practices such as platform design and operation. Secondly, perceived enjoyment is an important element that women are concerned about, which can attract female users and provide a more enjoyable fitness experience. On the one hand, through brand co-branding, supply chain integration and other ways to expand the product field, continue to update the product content, and make use of the platform's social features to organise online and offline group activities to enhance its enjoyment. On the other hand, by keeping up with the trend, popular hotspots, and so on, we continue to provide product stories, provide users with more connotative products, and promote marketing. Third, cross-border circle-breaking cooperation should be deepened to improve the efficiency of platform usage. On the one hand, the platform can cooperate with professional fitness coaches, nutritionists, and rehabilitators to increase the usage rate of the platform, improve the exposure rate of knowledge providers, and innovate the platform's business model; on the other hand, the platform can also cooperate with sports apparel sales, cultural and creative product sales, and wearable social bloggers, to enrich the content of the platform, and to achieve the simultaneous development of such products through sports themes such as camping, marathon, and cycling.

## 7.2 Limitations

Firstly, this study fails to adequately analyze the differences between groups in online fitness payment behaviors in the context of the “she-economy”. Future research could divide the sample according to “hedonic”, “comprehensive”, and “pragmatic” to identify group differences in the generation of online fitness payment behaviors. Secondly, perceived enjoyment positively influences satisfaction and continue using intention, which promotes online fitness payment behavior. However, the mediating effect was insignificant and could be examined more deeply in subsequent studies.
