# Supplementary material for: Effects and functional mechanisms of digital fitness platforms on online fitness payment behavior under the perspective of “She-economy”
Source: PLoS One. 2025 Mar 10;20(3):e0319246. doi: 10.1371/journal.pone.0319246 (PMC11892851; doi:10.1371/journal.pone.0319246)
Supplement: Attachment — Submitted filename: Response_to_Reviewers_auresp_3.doc [file pone.0319246.s004.doc]

**Dear Editor and reviewers:**

On behalf of all the contributing authors, I would like to express our gratitude for your helpful feedback on our article. Your comments have been invaluable in improving our work. According to your suggestions, we have revised the manuscript to make our results more convincing. We have highlighted all the changes made to the manuscript using tracked changes in red. We offer a detailed point-by-point response to your insightful questions and suggestions below.

**Editor:**

**Q1:** Please review your reference list to ensure that it is complete and correct.

**Response:** Thank you for your valuable suggestion. To ensure the reference list is complete and correct, we reviewed all references in the manuscript. Firstly, we checked all references to see whether the article was retracted. Secondly, we re-edited the reference format of the article by Endnote to make it consistent with the journal’s requirements. In summary, the order of references has not changed, and the changes in reference formatting we have marked in red in the manuscript.

**Reviewer #5:**

**Q1：**While the revised framework provides a stronger foundation for hypothesis formation, some aspects of the theoretical linkages could benefit from greater elaboration. A clearer articulation of the relationships between key constructs would help strengthen the coherence and rigour of the theoretical model.

Comment 1 – While I appreciate the efforts to include a conceptual framework that integrates the S-O-R paradigm and TAM, I remain unconvinced that the TAM model should serve as the stimulation phase in the S-O-R paradigm. The S-O-R framework traditionally relies on external stimuli (e.g., environmental cues) to influence internal states and subsequent behaviours, whereas TAM primarily focuses on users' perceptions and attitudes towards technology adoption. Thus, positioning TAM as the stimulation phase may not fully capture the environmental influences intended in the original S-O-R model.

**Respon**se: Thank you very much for your advice on the conceptual framework, which has been very enlightening. With your very professional knowledge, you gave us a perfect revision plan, which enhanced the completeness, logic, and coherence of the manuscript, and improved the quality of the manuscript, so thank you again. As you have stated, the use of the TAM model as the stimulus phase of the S-O-R paradigm in this study is controversial. The relationship between key concepts also needs to be further elaborated more clearly. Therefore, firstly, following your suggestion, I explored a hybrid model combining elements of the TAM and SOR models, with the TAM as part of the ‘O’. This enhances the conceptual model by recognising both environmental factors and users' internal evaluations. Secondly, the relationships between the key concepts in the framework have been analysed and elaborated in more detail, thus enhancing the coherence and logic of the article. The revisions have been highlighted in red in the manuscript. The specific changes are as follows:

In summary, the above provides an in-depth discussion of the role of women's fitness and the “She-economy” in digital consumer behaviour, as well as the theoretical basis of the “SOR theory” “TAM model”, the theoretical basis and the underlying context. Based on the above, a clear articulation of the relationships between key concepts will help to enhance the coherence and rigour of the theoretical model. The conceptual framework of this study is that digital fitness platforms act as a source of stimuli that generate perceivers after women's use, and these perceptions affect women's satisfaction and Continue using intention, which in turn affects online fitness payment behaviours. Therefore, the “S” framework of this study is an attribute of the digital fitness platform. A hybrid model is constructed by combining elements of the TAM and SOR models as the “O” framework. Females complete the “R” response by engaging in avoidance or proximity behaviours towards online fitness through the “S” and “O”. A well-constructed diagram (Fig 1) allows for a clearer and more organised presentation of these elements, enabling a more intuitive understanding of their connections and logical relationships, and providing a strong support for the following research to be carried out.

Fig 1. The conceptual framework

**Q2:** Comment 2 – The limitations section, as it stands, provides only a surface-level overview of the constraints faced in this study. To enhance the academic rigour and transparency of the manuscript, I recommend that this section be further developed with more meaningful insights. In particular, the limitations should reflect not only the methodological constraints but also theoretical and practical challenges encountered during the research process.

**Response:** Thank you for your valuable comments on the limitations section. Indeed, this section does not mention the theoretical and practical challenges, nor does it explicitly suggest directions in which future research could be conducted. I strongly agree with you that a more comprehensive account of the constraints of this study would add to the rigour and transparency of the manuscript. Therefore, firstly, I reflect on the possible generation of bias in sampling, including the range, number, and region of sample sources, and explore directions that could be improved in the future. Secondly, I reflect on the process of data collection and the results of data analysis, and explore the application of other methods in the future. Thirdly, we reflect on the group of people who pay for online fitness in the context of the ‘she economy’ and explore ideas for future research. Finally, I suggest a feasible direction for future research; the rapid development of technology brings different groups of people may have different impacts, resulting in consumption behaviours that are still worth exploring. At the same time, the large amount of Generative AI may also affect the quality of content in fitness platforms, and consumers' propensity to consume UGC and AIGC is still worth exploring. The specific changes are as follows:

Despite the new findings of this study, there are still some limitations in the empirical research process. First, the relatively small sample size and limited scope of this study may make the participants not fully representative of the broader population in the context of the “She economy”. Meanwhile, the cultural backgrounds of different countries and regions may also affect people's consumption behaviour on digital fitness platforms. Therefore, future research could adopt more diverse sampling methods, expand the sample coverage, and consider cross-cultural research in order to increase the generalisability of the findings and better reveal the complex dynamics between the two. Second, this study uses self-reported data collection methods, which may lead to deviations between reported data and the actual situation, thus affecting the accuracy of the findings. In addition, the mediating effects of satisfaction and continue using intention between perceived enjoyment and Online fitness payment behavior were not significant and deserve to be explored further. Therefore, future research could adopt data collection methods such as observation, longitudinal research methods such as tracking surveys, and qualitative research methods such as semi-structured interviews to reduce data bias and explore consumers' long-term behavioural patterns on digital fitness platforms in depth, and to further reveal the relationship between perceived enjoyment and other variables. Third, this study failed to adequately analyse the differences in online fitness payment behaviours between groups in the context of the “She economy”. Therefore, future research could divide the sample into “hedonic”, “comprehensive”, and “pragmatic” categories to clarify the group differences in online fitness payment behaviour.

With the advancement of technology, especially the rapid development of artificial intelligence, its application in digital fitness scenarios is bound to change our fitness habits and ways. Consumers will be able, on the one hand, to conduct comprehensive body diagnostic consultations and customise personalised fitness programmes through big language models in the fitness field, and on the other hand, they can use wearable devices such as VR and AR to learn fitness classes and carry out fitness exercises anytime, anywhere. However, the new technology may also creat a digital divide, causing exercise constraints for groups that are relatively insensitive to electronic information devices. At the same time, a large amount of Generative AI may also affect the quality of content in fitness platforms, and consumers' propensity to consume UGC and AIGC is still worth exploring.
