# Supplementary material for: Effects and functional mechanisms of digital fitness platforms on online fitness payment behavior under the perspective of “She-economy”
Source: PLoS One. 2025 Mar 10;20(3):e0319246. doi: 10.1371/journal.pone.0319246 (PMC11892851; doi:10.1371/journal.pone.0319246)
Supplement: Attachment — Submitted filename: Response_to_Reviewers_auresp_4.doc [file pone.0319246.s005.doc]

**Dear Editors:**

On behalf of all the contributing authors, I would like to express our gratitude for your helpful feedback on our article. Your comments have been invaluable in improving our work. According to your suggestions, we have extensively copyedited the English language usage and grammar our manuscript. We have highlighted all the changes made to the manuscript using tracked changes in red. We offer a detailed point-by-point response to your insightful questions and suggestions below.

**Editors:**

**Q1:** Ln 45, "Traditional thought, “soft beauty” is no longer the inherent label of women [8]. The Awakening of “Body Sense” [9], and The pursuit of “self-gratification, self transcendence and self-reading” is the “self-redemption” of women in the new era [10]" : It is unclear what this sentence means, and this would need to be clarified.

**Response:** Thank you very much for your comment. Indeed, the sentence was not expressed accurately enough, and the meaning was vague. Therefore, changes must be made. After checking the context of this sentence, we believe it is necessary to illustrate the increasing trend of women participating in fitness activities, which has drawn the fitness market’s attention to female consumers. Therefore, by modifying this sentence to “As it can be seen, female gym-goers have become the mainstay of the fitness population”, the logic of the passage can be made tighter and the language more condensed. The sentence will be more logical and concise.

**Q2:** ln 115, "Women’s fitness is a subordinate concept to fitness, with “body sensations” of pain, lightness, sweat, difficulty, heat, and being seen [9], emphasizing the important functions of body sculpting, emotional expression, and psychological construction [10]." : It is unclear what this sentence means, and this would need to be clarified.

**Response:** Thank you for your comment; it is your input that makes our research more rigorous. Indeed, the meaning of this sentence is not clear enough. Therefore, I think it is appropriate to revise this sentence to “Previous study reveals that, compared to men, women's fitness emphasizes more on the important functions of body sculpting, emotional expression, and psychological construction [27]”. This modification does not change the semantic meaning and makes the article clearer.

**Q3:** ln 118, "In the context of China’s socialist culture, women’s fitness has been influenced by the group’s body-shaping aspirations, the indulgence of social visual experience, and the advancement of the national fitness strategy, resulting in a number of research findings" : It is unclear what this sentence means, and this would need to be clarified. This sentence also needs to be supported by an appropriate reference.

**Response:** Thank you for your comment. This sentence is indeed inaccurate and also needs to be supported by an appropriate reference. Therefore, I modify this sentence to read “Women's fitness in China has been influenced by a combination of social culture [28] and national strategies [29], resulting in a number of issues with local characteristics that have attracted the attention of many scholars”, and added 2 references. The advantage of this is that it does not change the semantic meaning and is straightforward to express, so that readers can understand our work more clearly.

**Q4:** Ln 124, "women’s fitness reconstruction of the gender order" : It is unclear what this sentence means, and this would need to be clarified.

**Response:** Thank you very much for your comments. This sentence is not clear enough. Therefore, we revise the sentence to read “the function of fitness in promoting women's social status”. This isn’t change the meaning of the sentence and make the article clearer.

**Q5:** Ln 246 - 250: It would be helpful if the authors briefly describe the differences, rather than only stating there was a difference.

**Response:** Thank you very much for your comments. Yes, a brief description of the differences would be more helpful for readers to understand our work. The modifications are as follows:

Zhan H [63] took college students as research subjects to explore their continue using intention online teaching platforms and found that perceived usefulness had a more pronounced effect on male students' continue using intention. Women college students' continue using intention the platform was more influenced by social factors. Tan H et al. [64] explored the effects of men’s and women’s student satisfaction and continue using intention with teachers online teaching, and the results showed that there were significant differences. Men students paid more attention to the external environment in their teachers' online teaching, while women students were more concerned with the learning outcomes and the quality of learning.

**Q6:** ln 539- ln 548: This seems to be the author's opinions only, and are unsupported by primary literature and as such would require clarification or revised to be removed.

**Response:** Thank you again for your comments. The passage does lack support from the literature. Therefore, I have added references. The modifications are as follows:

The perceived usefulness of only female users of digital fitness platforms has little impact on their satisfaction. The underlying reasons for this are as follows: firstly, from a physiological point of view, women have lower extrinsic explosiveness and intrinsic testosterone hormones, which are important reasons for slow muscle growth [91]. Thus obtaining fewer positive incentives and lagging in the perceived usefulness for the software. Secondly, based on the compensation theory for an explanation, when women do not feel that the digital fitness platform can improve the quality of life, they will have a sense of frustration and look for new ways to make up for the loss; the enjoyment feeling appears just to make up for the lack of usefulness, thus forming compensation [92].

**Q7:** ln 575-ln 577: This seems to be the author's opinions only, and are unsupported by primary literature and as such would require clarification or revised to be removed.

**Response:** Thank you very much for your comments. Indeed, the passage lacks support from the literature. Therefore, I have added references. The modifications are as follows:

Women may spend impulsively because of a strong excitement about the platform service, and are not overly concerned about satisfaction the digital fitness platform [98]. This explains why some women impulsively spend money on signing up for contests or events on platforms just to win fun prizes [99].

**Q8:** ln 585-592: This seems to be the author's opinions only, and are unsupported by primary literature and as such would require clarification or revised to be removed.

**Response:** Thank you very much for your comments. Indeed, the passage lacks support from the literature. Therefore, I have added references. The sentence “Meanwhile, focusing too much on the hedonic experience may affect the achievement of the actual fitness effect to a certain extent” lacks support from literature and seems to be promoted based on individuals’ life experiences. Therefore, we consider that it would be more rigorous to delete this sentence. The modifications are as follows:

This further reflects the consumption characteristics of women [100], who tend to be easily attracted to services and products with novel and interesting designs when using digital fitness platforms [101]. They may impulsively consume because of an interesting challenge activity, a personalized training plan, or an attractive interface design on the platform [98]. However, this kind of impulsive consumption may also bring some problems, such as buying services or products that are not suitable for them because of their momentary interest, leading to waste [102].

**Q9:** ln 606 : It is unclear what this sentence means, and this would need to be clarified.

**Response:** Thank you very much for your comment. The sentence was indeed not clearly expressed. Therefore, I have rearranged the sentence here. The modifications are as follows:

This is consistent with the findings of the Hargreaves J’s study [106].

**Q10:** ln 634-635, "However, it may be due to reasons such as women's presentation of fitness results being poorer than men's": The article that has been referred to support this statement is not an appropriate one to support this. It is also further unclear what this means or what is meant by fitness results.

**Response:** Thank you very much for your advice. Yes, the sentence needs to be expressed clearly enough and have a logical relationship before and after. The reference citation is also inappropriate. Therefore, I have removed this sentence as well as this reference. The following sentence has been modified, but the semantic meaning remains the same. The purpose of this is to make the preceding and following content more logically coherent. The modifications are as follows:

However, no significant relationship was found between the usefulness of the platform and women's satisfaction, implying that women care less about the usefulness of the platform than about enjoyment and ease of use.

Several references were added and deleted as a result of the manuscript. Thus the reference order of the revised manuscript has changed compared to the original manuscript. We have marked the changes in red in the manuscript with track changes and listed the comparison of the reference order of the two manuscripts in the table below.

| Number | |
| --- | --- |
| In the Original Manuscript | In the Revised Manuscript |
| [1]-[7] | [1]-[7] |
| [8]-[9] | deleted |
| [11]-[29] | [8]-[26] |
| [10] | [27] |
| - | [28],[29] |
| [30]-[90] | [30]-[90] |
| - | [91],[92] |
| [91]-[95] | [93]-[97] |
| - | [98],[99] |
| [96] | [100] |
| - | [101],[102] |
| [97]-[101] | [103]-[107] |
| [102] | deleted |
| [103] | [108] |
